# The Prevalence of and Predisposing Factors for Late Atrial Arrhythmias after Transcatheter Closure of Secundum Atrial Septal Defects in Children

**DOI:** 10.3390/jcm12113717

**Published:** 2023-05-28

**Authors:** Tariq Abu-Tair, Claudia Martin, Christiane M. Wiethoff, Christoph Kampmann

**Affiliations:** 1 Department of Pediatric Cardiology, Friedrich-Alexander-University Erlangen-Nürnberg, 91054 Erlangen, Germany; 2 Department of Congenital Heart Disease, Centre for Diseases in Childhood and Adolescence, University Medicine Mainz, 55131 Mainz, Germany; martin.claudia@me.com (C.M.); christiane.wiethoff@yahoo.de (C.M.W.); kampmann@uni-mainz.de (C.K.)

**Keywords:** ASD closure, interventional cardiology, pediatric, arrhythmia

## Abstract

Background: A 24 h Holter study in children after transcatheter secundum ASD (ASD II) closure was conducted to detect the prevalence of defects and/or device-related late atrial arrhythmias (LAAs). ASD II closure with an Amplatzer septal occluder (ASO) is an established procedure. Little is known about LAAs after device implantation. Methods: The eligible participants were children who had undergone ASO implantation, with a follow-up of ≥5 years, as well as one pre- and at least one post-procedural Holter ECG. Results: In total, 161 patients (mean age: 6.2 ± 4.3 years), with a mean follow-up of 12.9 ± 3.1 years (range 5–19), were included. A median of four Holter ECGs per patient were available. LAAs occurred before intervention in four patients (2.5%), and it was peri-interventional in four patients (2.5%), sustained in three patients (1.9%), and developed in three patients (1.9%). In patients with pre- and peri-interventional LAAs, the Qp/Qs ratio was higher (6.4 ± 3.9 vs. non-AA: 2.0 ± 1.1 (*p* = 0.002)) and the IAS/ASO ratio was lower (1.18 ± 0.27 vs. non-AA: 1.7 ± 0.4 (*p* < 0.001)). The patients with LAAs differed from those without LAAs in their Qp/Qs (6.8 ± 3.5 vs. 2.0 ± 1.3; *p* < 0.0001) and IAS/ASO ratios (1.14 ± 0.19 vs. 1.73 ± 0.45; *p* < 0.001). The patients with LAAs had a Qp/Qs ratio ≥2.94:1, and those who developed LAAs had an IAS/ASO ratio <1.15. Conclusions: LAAs occurred in 1.9% of patients and were sustained in another 1.9% of patients but persisted in those with large shunt defects and large occluders in relation to the atrial septal length. The predisposing factors for LAAs after ASD closure were a high Qp/Qs ratio, pre-existing atrial arrhythmias, and a low IAS/ASO ratio.

## 1. Introduction

Secundum atrial septal defects (ASDs) are one of the most common congenital cardiovascular defects with an incidence of 6–10 per 10,000 live births, which may require intervention [1]. During the past ~25 years, the closure of centrally located ASDs has moved from a surgical approach to a well-established percutaneous transcatheter therapy, while defects outside the fossa ovalis are still in the domain of open-heart surgery. The surgical repair of an atrial septal defect has negligible mortality, but it is associated with morbidity, discomfort, pain, and higher costs [2]. As an alternative to surgery, in 1976, King et al. described the first transcatheter closure of an ASD [3]. Since then, different devices have been developed and made available [4]; some have remained on the market, while others have been withdrawn [5,6,7]. One of the devices to remain on the market is the Amplatzer septal occluder (ASO), which was first introduced in humans in 1995 [8] and has been implanted more than 200,000 times. It has been shown that transcatheter closure results in the normalization of cardiac size and function [9,10,11], and short- and intermediate-term studies have shown the ASO to be safe and effective [12,13,14,15]. Furthermore, it has been demonstrated that the transcatheter mortality and morbidity in this procedure are lower than the surgical approach [2,16,17,18,19]. Nevertheless, due to the widespread use of this device, a great variety of adverse events have been reported, e.g., device embolization, erosion and cardiac perforations, thromboembolic complications, device infection, residual defects, and others, as discussed in the literature [20]. One of the major concerns about the ASO is its stiffness [21] and the weight of the occluder. Once the device is covered with the endothelium and changes its morphological configuration, it is thought to become even stiffer [22]. It is conceivable that the stiffness of the occluder and its weight might contribute to the occurrence of late atrial arrhythmias (LAAs). Nevertheless, the ASO is the longest-lasting device on the market. Several reports concerning the safety and efficacy of the ASO have been published in recent years, but reports of arrhythmias after implantation are rare and such studies are usually not conducted solely in children and usually take place in a follow-up period up to five years after implantation [23,24,25]. Several publications have reported on peri-interventional arrhythmias, mainly complete AV blocks, usually resulting in the removal of the device [26,27], and there have been case presentations of the development of late complete AV blocks after ASO implantation [28]. Therefore, the aim of this Holter ECG study was to analyze the outcome of a large cohort of children after ASO implantation with respect to the persistence of arrhythmias and development of late arrhythmias five and more years after implantation, related to the defect itself and/or the implanted device.

## 2. Materials and Methods

Between July 1996 and July 2021, a total of 638 patients underwent percutaneous closure of an atrial septal defect in a single center.

Those eligible for these analyses were patients with a successful complete ASD closure (confirmed by echocardiography) using one ASO. The routine follow-up consisted of echocardiographic evaluation and a twelve-lead ECG at one, three, and six months and annually after closure. In each patient, there was at least one Holter ECG study of a minimum 20 h duration before closure, peri-procedural ECG recordings, and at least one follow-up Holter ECG study with a minimum of 20 h recorded. The Holter ECGs have been analyzed exclusively by specialists in pediatric cardiology. The follow-up needed to exceed at least 5 years. Holter ECGs were routinely performed in any patient with suspicion of atrial arrhythmias pre- and post-interventionally.

Cardiac catheterization was always performed in conscious sedation with S-ketamine and midazolam to avoid hemodynamic alterations due to mechanical ventilation or lowered blood pressure. Additionally, hemodynamic measurements were performed before inserting the TEE probe for echocardiographic guidance to prevent respiratory and consecutive hemodynamic changes.

Procedural information regarding the Qp/Qs shunting by oximetry calculated using Fick’s formula, the echocardiography-estimated interatrial septal length at the level of the greatest ASD diameter in the subcostal four-chamber view, and the relationship between the interatrial septal length and the left atrial occluder diameter (IAS/ASO ratio) were evaluated.

Patients older than 18 years of age at implantation, in whom a device other than the Amplatzer septal occluder or more than one device had been implanted and those who had any residual shunt, complex congenital heart disease, or any kind of cardiac surgery before or during follow-up, were excluded. Additionally, patients with pulmonary hypertension were excluded. Pulmonary hypertension was defined as an invasively measured mean pulmonary artery pressure equal to or exceeding 25 mmHg, which was not related to left–right shunting.

Four hundred and seventy-seven patients were excluded from this study. Ninety-seven patients were older than 18 years of age at implantation. In 153 patients, devices other than the Amplatzer septal occluder were implanted. In four patients, more than one device (up to three devices from different manufacturers) was implanted. Twenty-eight patients were excluded due to residual shunt, complex congenital heart disease, or any kind of cardiac surgery before or during follow-up. One hundred and thirty-four patients were excluded due to follow-up time being less than 5 years. In addition, 231 patients were excluded from the study due to incomplete follow-up data and loss to follow-up. Eleven patients with pulmonary hypertension were excluded; one of these patients needed a fenestration of the implanted device. Seventy-six of the patients fulfilled more than one exclusion criteron.

Atrial arrhythmic events were classified as supraventricular tachycardia, constant ectopic atrial rhythm, AV dissociation, atrial flutter or fibrillation, AV blocks, and sick sinus syndrome. Supraventricular premature beats were excluded due to their frequent occurrence even in healthy patients. As specific ECG parameters are age-dependent, judgements were made according to the normalized values presented by A. Davignon.

Statistical analyses were conducted using SPSS (IBM, Armonk, NY, USA, Ver. 28 for MAC OS). The data are expressed as a frequency or percentage for nominal variables and as the mean ± SD for continuous variables. Differences among the outcomes between the groups were analyzed using unpaired Student’s *t*-tests. If the distribution of the variable was not normal according to the Wilcoxon–Shapiro test, the Mann–Whitney U test was used. All tests were two-sided. ROC analyses were conducted to detect the shunt ratio amount (Qp/Qs) and/or the IAS/ASO ratio when arrhythmias occurred. The patients were grouped according to their shunt volume into group I with a Qp/Qs ratio < 2.5:1 and into group II with a Qp/Qs ratio ≥ 2.5:1. Longitudinal differences were calculated using the log-rank (Mantel–Cox) test. ANOVA testing was used to describe the factors influencing the occurrence of late arrhythmias. A *p*-value < 0.05 was considered significant.

## 3. Results

In total, 161 patients were eligible and fulfilled the inclusion criteria. Of these, 65 patients (40.4%) were male. The mean age of the patients at closure was 6.2 ± 4.3 years, with a range from 0.04 years to 16.6 years. The Qp/Qs ratio ranged from 1.0 to 7.0 with a mean of 2.2 ± 1.6. The IAS/ASO ratio ranged from 1.0:1 to 3.0:1 with a mean of 1.7 ± 0.5. There were no differences regarding age, shunt, IAS/ASO ratio, or available follow-up data between the sexes.

The mean follow-up was 13.2 ± 3.04 years, ranging from 5.0 to 19.2 years. In total, 408 Holter ECGs were available with a median of three post-procedural ECGs (range 1–7) per patient.

In total, 4 of the 161 children (2.5%) had documented atrial arrhythmias before closure, with 1 patient having a first-degree AV block, 2 patients having a constant ectopic atrial rhythm, and 1 patient having a constant AV dissociation. Those patients with pre-existing arrhythmias did not differ in age from those without (5.1 ± 5.1 vs. 6.27 ± 4.27 years, *p* = 0.6), but they differed in shunt volume (8.25 ± 4.6 vs. 2.03 ± 1.2; *p* < 0.0001). After closure, one of the two patients with an ectopic atrial rhythm converted to a normal sinus rhythm. In the other three patients, atrial arrhythmia was sustained.

In 4 out of the 161 patients (2.5%), atrial arrhythmias developed during the intervention. One patient developed supraventricular tachycardia (with the need for pharmacological conversion), one developed transient sinus arrest, one developed a temporary complete AV block, and one developed an ectopic atrial rhythm. Only the ectopic atrial rhythm persisted for a short time after the procedure. The patients with peri-interventional arrhythmias were younger (3.7 ± 1.3 vs. 6.3 ± 4.3 years; *p* = 0.018) and had higher shunt volumes (4.0 ± 2.3 vs. 2.1 ± 1.6; *p* = 0.04). During long-term follow-up, none of the patients with temporary arrhythmias showed a recurrence of the rhythm disturbance.

In total, 8 out of the 161 (5%) patients showed both pre- and peri-interventional atrial arrhythmias. The patients with pre- and peri-interventional arrhythmias did not differ in age (4.4 ± 3.6 vs. 6.34 ± 4.3 years; *p* = 0.2) but did differ in shunt volume (4.4 ± 3.9 vs. 2 ± 1; *p* < 0.0001) and in the number of Holter ECGs during follow-up (3.5 ± 1.2 vs. 2.5 ± 1 Holter ECGs; *p* = 0.048). Out of those 8 patients with pre- and peri-interventional arrhythmias, the arrhythmias were sustained in 3 patients from the total of 161 patients. In one patient, the I° AV block persisted and, in two patients, the ectopic atrial rhythm persisted.

Aside from those three patients in whom arrhythmias were sustained during follow-up, another three patients developed atrial arrhythmias in a follow-up period of at least 5 years. Again, the most common were ectopic atrial rhythms in two patients, along with an I° AV block in one patient. Those three patients had a Qp/Qs ratio of 4.5 ± 4 and an IAS/ASO ratio of 1.16 ± 0.1.

None of the patients with atrial arrhythmias received any antiarrhythmic treatment except the patient with SVT during the procedure who received pharmacological conversion.

The 155 patients without any LAA events, compared to those who showed sustained atrial arrhythmias, did not differ in age at closure (6.3 ± 4.3 vs. 5.6 ± 4.2 years; *p* = 0.71), but they did differ in shunt volume (2.0 ± 1.3 vs. 4.8 ± 2.5; *p* < 0.0001) and in IAS/ASO ratio (1.73 ± 0.45 vs. 1.14 ± 0.19; *p* = 0.002).

Due to the conceivable influence of shunt volume, the patients were grouped arbitrarily according to their Qp/Qs ratio into two groups: patients with a shunt volume < 2.5:1 (group I) and patients with a shunt volume ≥ 2.5:1 (group II) (Table 1). The patients with larger shunts received transcatheter closure at significantly younger ages and had lower IAS/ASO ratios. Patients in both groups had the same duration of follow-up and did not differ in the number of post-procedural Holter ECGs.

Only in the subgroup of 30 patients with a Qp/Qs ratio ≥ 2.5:1 (group II) were persisting atrial arrhythmias observed (6/30 patients; 20%); here, three of the four pre-existing and three of the four peri-interventional atrial arrhythmias were noticeable. In the two patients with both pre- and peri-interventional atrial arrhythmias with a shunt volume below 2.5:1 (2/131 patients; 1.5%), arrhythmias were not sustained and were completely resolved after the procedure. Of the 131 patients with a Qp/Qs ratio < 2.5:1, all of the patients were in sinus rhythm over a mean follow-up period of 13 years, proven by a median of 2 Holter ECGs per patient, whereas in the 30 patients with a Qp/Qs ratio ≥ 2.5:1, 24 patients were in sinus rhythm and 6 patients developed or sustained atrial arrhythmias, proven by a median of 3 Holter ECGs per patient (*p* < 0.0001).

An ROC analysis was performed to detect the shunt volumes and IAS/ASO ratios at which the atrial arrhythmias were sustained and/or occurred during follow-up. No persisting atrial arrhythmias occurred in patients with a shunt volume < 2.94:1 (100% sensitivity, 92% specificity) and with an IAS/ASO ratio > 1.15:1 (63% sensitivity and 93% specificity). Those three patients who developed atrial arrhythmias during follow-up had a median age of 2.5 (0.04–6) years, a median Qp/Qs ratio of 4.4:1 (3.2–7), and a median IAS/ASO ratio of 1.1 (1.0–1.15).

The predisposing factors for the development of late arrhythmias after interventional ASD closure were the Qp/Qs ratio (F = 69.2; *p* < 0.0001), pre-existing atrial arrhythmias (F = 35.4; *p* < 0.0001), and the IAS/ASO ratio (F = 9.8; *p* < 0.002).

## 4. Discussion

Transcatheter closure of secundum ASDs is almost ubiquitously available; compared to surgery, it has the same immediate efficacy and safety but lower morbidity [18,19]. Even in infants below 1 year of age, percutaneous closure is feasible [29,30,31]. In elective cases, the optimal age for surgery for ASD is usually ~4–6 years; here, the operative risk, mortality, morbidity, and long-term outcome are well balanced [32,33]. Therefore, in some centers, the timing for transcatheter closure is derived from the surgical source. However, in contrast to surgery for atrial septal defects, long-term data of transcatheter techniques are rudimentary; yet, in children, they are of utmost importance because children have the longest life expectancy, with a lifelong device in place [25,34,35,36,37,38,39,40,41]. Therefore, follow-up of these patients is mandatory. The long-term outcome of surgical ASD closure is excellent survival with low morbidity and mortality proven in studies up to 21–33 years post-surgery, but during surgical procedures, the risk of arrhythmias is higher in surgery than in interventional closure [32,42]. ASD itself is associated with an increased risk of arrhythmias and conduction disorders due to right-sided volume overload and electrical remodeling [43]. Therefore, the risk of arrhythmias increases with late repair, an increased shunt size, and other factors such as pulmonary hypertension and comorbidities.

On the other hand, the incidence of arrhythmias decreases following ASD closure. Sinus node dysfunction is a rare complication especially in older surgical repair techniques [44].

Nevertheless, surgical ASD closure improves AV conduction, decreases the AV nodal refractory period, and improves sinus node function, probably by suppressing right-sided volume overload. However, sinus node function can be lost as a result of the surgical procedure [35].

The Amplatzer septal occluder is the most widely used atrial septal occluder, and it has been approved since 1996. Some of the major advantages of this device are the small introduction sheaths, full retrievability, almost 100% occlusion, and a very low thromboembolic complication rate in children [30]. It is speculated that one of the major concerns about this device might be, once fully deployed, its stiffness, which becomes even greater after complete endothelialization, along with the relatively large amount of material and the weight of the device itself. This could contribute to the development or occurrence of late arrhythmias after the successful closure of the defect. Therefore, this study was solely focused on the Amplatzer septal occluder.

Holter ECG as a tool to monitor atrial arrhythmias is in widespread use, but the detection rate is estimated to be about 5%, and the procedure should only be performed if there is a high probability for arrhythmias [45]. In addition, studies have shown that decisions based on Holter ECG have led to extended hospitalization but with no effective yield [46]. On the other hand, Holter ECG should be performed in these cases due to the frequent occurrence of atrial arrhythmias in this patient group which should be monitored as depicted in multiple studies [32,40,43,47]. Even though the detected arrhythmias are initially benign and require no further treatment, they are possible predictive parameters for further atrial arrhythmias or atrial flutter or fibrillation later in life [48,49]. This has to be considered especially if electrogeometric remodeling does not reverse over time [43].

This is the first report of a long-term follow-up of solely pediatric patients after successful closure of an isolated atrial septal defect using the Amplatzer device in a substantial number of patients. In general, it can be seen that during a mean follow-up of 13 years after implantation, atrial arrhythmias occurred but were generally benign, as shown by other authors in small studies [50]. No patient developed or showed complex atrial or atrioventricular arrhythmias. However, at young ages, 10% of patients with a large shunt volume above a Qp/Qs ratio of 3:1 already had pre-existing atrial arrhythmias in terms of AV dissociation and ectopic atrial rhythms or I° AV block. Pre-existing arrhythmias have been described in previous studies according to shunt volume, leading to right atrial enlargement and resulting in adverse electrogeometric remodeling. This leads to the recommendation to close atrial septal defects at an early age before arrhythmias occur as electrogeometric remodeling does not reverse in contrast to morphological remodeling once the defect is closed [43]. Even during the procedure, the susceptibility of developing arrhythmias seemed to be higher in patients with large shunt volumes and those with the need for larger devices; however, the patients who developed peri-procedural arrhythmias were generally younger than those with uneventful procedures. Nevertheless, the peri-procedural arrhythmias were more complex and harmful, such as supraventricular tachycardia, which needed pharmacological conversion, and a complete AV block, which required pacemaker support for 65 min, but both were transient.

During the follow-up of up to 19 years, three additional patients developed atrial arrhythmias. Two patients developed ectopic atrial rhythms, and one patient developed I° AV block without aggravation during repeated Holter ECGs. These three patients were free of atrial arrhythmias in the pre-implantation Holter ECGs and in up to nine twelve-lead regular surface ECGs. Therefore, it is conceivable that, in these patients, atrial arrhythmias developed after the percutaneous closure of the ASD. In addition to having shunt volumes above 2.5:1, these patients had the largest occluders in relation to the interatrial septal length with an IAS/ASO ratio below 1.15:1 (median 1.1:1); furthermore, with a median age of 2.5 years, they were relatively young. Corresponding to our results, Wang et al. recognized an increased risk of AV blocks in patients with a large occluder size in relation to the septal length and a young age [51]. The “rule of thumb” is that occluder sizes above 1 mm/kg of body weight are assumed to be high-risk procedures; yet, they are becoming increasingly more frequent according to the experience of interventionalists [52]. Nevertheless, the IAS/occluder ratio offers a more accurate risk stratification for the occurrence of late atrial arrhythmias. It is remarkable that despite the growth of surrounding areas during aging and electrogeometric atrial remodeling, late atrial arrhythmias can still occur, where a large occluder size is shown to be a risk factor [47]. Studies regarding Amplatzer erosions have directed attention to the atrial septal size and “landing zone” of closure devices [21,22,53]. In addition to the occurrence of these erosions, areas with high pressure on the atrial wall due to the device’s position were identified, which could be assumed as the origins of atrial arrhythmias. In these cases, in addition to monitoring for erosions, the occurrence of atrial arrhythmias should be monitored over long-term follow-up.

The percutaneous transcatheter closure of atrial septal defects using the Amplatzer septal occluder is a safe and effective therapeutic option. However, the presented data suggest that, to avoid late-onset atrial arrhythmias, device closure should be performed before atrial arrhythmias occur, corresponding to the evidence given in the literature [43]. Occluder-related late atrial arrhythmias are benign and rare (with an incidence of 1.9%), affecting only those patients with high shunt volumes above a Qp/Qs ratio of 2.5:1 and large occluders in relation to the interatrial septal length. Furthermore, it has to be considered that, even below the age of 6 years, patients with large shunt volume atrial septal defects may have atrial arrhythmias, which may persist after successful transcatheter defect closure.

## 5. Limitations

Our study has several limitations, which were mainly based on its retrospective design and small sample size. The presented results need to be confirmed in further multicentric trials. Some patients may have asymptomatic undiagnosed arrhythmias which could be missed by using only Holter ECG exams. Nevertheless, the authors assume that Holter ECGs are an easily available, non-invasive tool for the screening of even asymptomatic patients after ASO. We assume that lifelong screening for arrhythmias should be performed, as our data suggest.

Furthermore, the interatrial septal length was estimated only in the subcostal four-chamber view at the level of the largest ASD diameter. Protruding closure devices could induce pressure on any nearby cardiac structure. Therefore, the geometry of the interatrial septum contains multiple areas possibly altered by the occluder, leading to atrial arrhythmias. Recent studies regarding occluder erosions have identified several regions where oversized closure devices could induce atrial arrhythmias, which should be observed and evaluated regarding the impact of the occurrence of atrial arrhythmias after ASD closure.

## 6. Conclusions

Our data suggest a low incidence of mostly benign late atrial arrhythmias, which is in agreement with short- and intermediate-term outcome studies. Especially in large defects with high shunt volumes, arrhythmias could be frequently detected pre-intervention. In addition to pre-existing arrhythmias, a high shunt volume above a Qp/Qs ratio of >2.5/1, a young age at intervention, and large occluder sizes with an IAS/occluder ratio < 1.15:1 are risk factors for the occurrence of late atrial arrhythmias.

## Figures and Tables

**Table 1:** Patient data, grouped according to shunt volume.

	Group I (Qp/Qs < 2.5)	Group II (Qp/Qs ≥ 2.5)	*p*
Number	131	30	
Age (years ± SD)	6.7 ± 4.3	4.1 ± 3.7	<0.001
Qp/Qs (±SD)	1.6 ± 0.4	4.6 ± 2.7	<0.0001
IAS/ASO (±SD)	1.8 ± 0.4	1.2 ± 0.3	<0.0001
Follow-up (years ± SD)	12.9 ± 3.1	13.1 ± 2.7	0.78
No. of Holters (*n* ± SD)	2.46 ± 1.07	2.87 ± 1.04	0.06
Patients with persisting LAAs	0	6	<0.001

Abbreviations: age: age at percutaneous closure; Qp/Qs ratio: left-to-right shunt quantification measured by oximetry; IAS/ASO ratio: the interatrial septal length to the left atrial external dimension of the implanted device estimated by echocardiography from a short-axis view; no. of Holters: number of Holter ECGs available post implantation; patients with persisting LAAs: number of patients with persisting atrial arrhythmias for more than 5 years of follow-up; (±SD): plus/minus the standard deviation.

## Data Availability

T.A.-T. and C.K. had full access to all the data in the study and take responsibility for the integrity of the data and the accuracy of the data analysis. The data presented in this study are available from the corresponding author upon request. The data are not publicly available due to privacy reasons (medical data).

## Abbreviations

ASD II	Secundum atrial septal defect
ASO	Amplatzer septal occluder
LAA	Late atrial arrhythmia
IAS	Interatrial septal length
IAS/ASO ratio	Interatrial septal length to the left atrial external device diameter of the ASO
Qp/Qs ratio	Ratio of pulmonary to systemic blood flow, estimated from oximetry without oxygen supplementation
